# Comparison of entropion outcome with and without intervention in Romane and Ile de France sheep breeds

**DOI:** 10.1002/vms3.1317

**Published:** 2023-10-30

**Authors:** Hossein Esmaeili, Amir Hossein Alinejad, Mona Hamedi, Sergio Villanueva‐Saz, Marta Ruiz de Arcaute, Diana Pérez, Juan Ibañez, Delia Lacasta

**Affiliations:** ^1^ Department of Microbiology and Immunology Faculty of Veterinary Medicine University of Tehran Tehran Iran; ^2^ Department of Animal Pathology Veterinary Faculty University of Zaragoza Zaragoza Spain; ^3^ Instituto Agroalimentario de Aragón‐IA2, Universidad de Zaragoza‐CITA Zaragoza Spain; ^4^ Department of Ophthalmology Hospital Universitario Lozano Blesa Zaragoza Spain

**Keywords:** entropion, Ile de France, Iran, Lamb, Romane

## Abstract

**Background:**

Congenital entropion is the most frequent ocular disorder in newborn lambs of certain sheep breeds, which, if not treated, can result in complete blindness and death due to starvation.

**Objectives:**

The aims of this study were to compare the spontaneous healing of entropion in two breeds and assess the outcome of cases with and without therapeutic intervention.

**Methods:**

A total of 158 entropion cases (119 Ile de France and 39 Romane) were investigated, and swab samples were collected from the cornea and conjunctiva of 73 of the affected lambs for bacteriological investigation. In addition, an ocular intervention was carried out in 123 affected animals.

**Results:**

The Romane breed developed entropion at an average age of 7 days compared to the Ile de France, which developed it at an average age of 1 day. Likewise, significant differences were found between bilateral and unilateral involvement in both breeds. Meanwhile, 22.1% of cases recovered spontaneously, and the highest rate of spontaneous recovery without intervention was observed in the Romane breed (66%). Bacteria isolated from ocular samples included *Staphylococcus* spp. (42.5%), *Bacillus* spp. (21.9%), *Trueperella pyogenes* (13.7%), *Corynebacterium* spp. (12.3%) and *Escherichia coli* (9.6%).

**Conclusions:**

The results of the study showed that the onset time of entropion, bilateral involvement, the severity of the process and the need for re‐treatment were higher in the Ile de France breed than in the Romane breed. Likewise, the Romane breed showed a higher degree of spontaneous recovery of entropion.

## Graphical abstract

We described a cross‐sectional study of congenital entropion in sheep including 158 cases (119 Ile de France and 39 Romane), evaluating bacteriological investigation and an ocular intervention. The most common bacteria isolated were *Staphylococcus* spp. (42.5%), *Bacillus* spp. (21.9%), *Trueperella pyogenes* (13.7%), *Corynebacterium* spp. (12.3%) and *Escherichia coli* (9.6%). The results of the study showed that the onset time of entropion, bilateral involvement, the severity of the process and the need for re‐treatment were higher in the Ile de France breed than in the Romane breed. Likewise, the Romane breed showed a higher degree of spontaneous recovery of entropion.

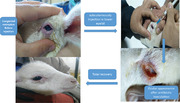

## 1 INTRODUCTION

Entropion is an ocular pathology characterized by inward rotation of the eyelid. The skin of the eyelid and eyelashes come into contact with the eye, generating continuous rubbing of the ocular surface and cornea, in an early manner and causing red eye, constant discharge, corneal thinning, risk of infection and, in severe cases, eye perforation, endophthalmitis and loss of vision. Entropion can be unilateral or bilateral, and the affection of the lower eyelid is much more common than that of the upper eyelid in most species (Warwick & Berry, 1962). In sheep, it is almost always limited to the lower eyelid (Moore & Whitley, 1984). In neonate lambs, this condition leads to severe keratoconjunctivitis, which, if not treated, can result in corneal ulceration, corneal opacity, suppurative panophthalmitis and eye loss. If both eyes are affected, this can cause complete blindness and death due to starvation (Scott, 2015).

Congenital entropion has been described in several sheep breeds, including Columbia, Oxford, Cheviot, Suffolk, Border Leicester, Rambouillet, Texel and Charollais (Green et al., 1995), and has been reported to affect up to 80% of lambs in a flock (Cameron et al., 2005; Wyman, 1983). The congenital form is considered the most frequent ocular disorder of newborn lambs (Shaw‐Edwards, 2010). However, the acquired form of entropion may be developed as a result of severe dehydration, eyelid scarring, trauma and loss of retrobulbar fat in debilitating diseases (Shaw‐Edwards, 2010). Entropion is considered an inherited disorder in sheep for which a polygenic inheritance pattern applied (Green et al., 1995). Heritability of this trait was estimated to be 0.21 and 0.17 for Columbia and Suffolk breeds, respectively (Sakul & Kellom, 1997). In this sense, the importance of genetic selection is crucial to reduce the incidence of entropion (Green, 1991; Taylor & Catchpole, 1986).

There are different results reported concerning entropion outcome with and without medical intervention. Some authors said that most cases of entropion resolve spontaneously or with minor medical intervention (Warwick & Berry, 1962). However, some researchers believe that intervention is needed in entropion cases because of economic and animal welfare reasons (Green et al., 1995; Sakul et al., 1996).

A variety of techniques such as Hotz‐Celsus, excising a crescent of skin close to the lid margin, subcutaneous injection in the eyelid or pinching and wound clip have been reported to correct entropion (Cameron et al., 2005). However, subcutaneous injection of antibiotics in the eyelid is the most common technique applied in farms because of its simplicity and efficiency.

The aims of this study were (1) to compare spontaneous healing of entropion in Romane and Ile de France breeds, (2) to assess the outcome of cases with and without therapeutic intervention and (3) to determine the presence of bacteria in cases of infection.

## 2 MATERIALS AND METHODS

### 2.1 Animals

A total of 158 entropion cases in lambs (119 Ile de France and 39 Romane) from different farms of Iran were investigated during 2018–2019 period. The severity of the clinical cases ranged from no ocular lesion to keratitis, corneal ulcers and blindness. General information about each lamb was recorded based on a checklist that included the following items: breed, litter size, age, affected eyes, date of onset of entropion, the presence of keratitis and evolution and outcome of entropion.

### 2.2 Clinical assessment

**FIGURE 1:**
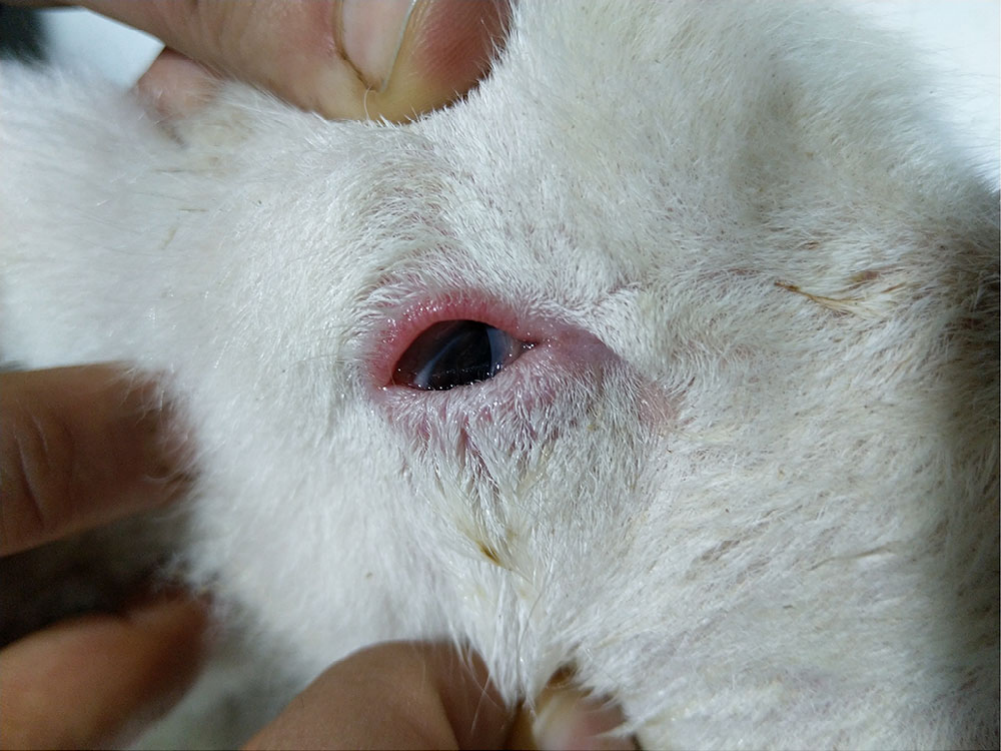
Mild entropion picture with the absence of ocular redness or tearing.

**FIGURE 2:**
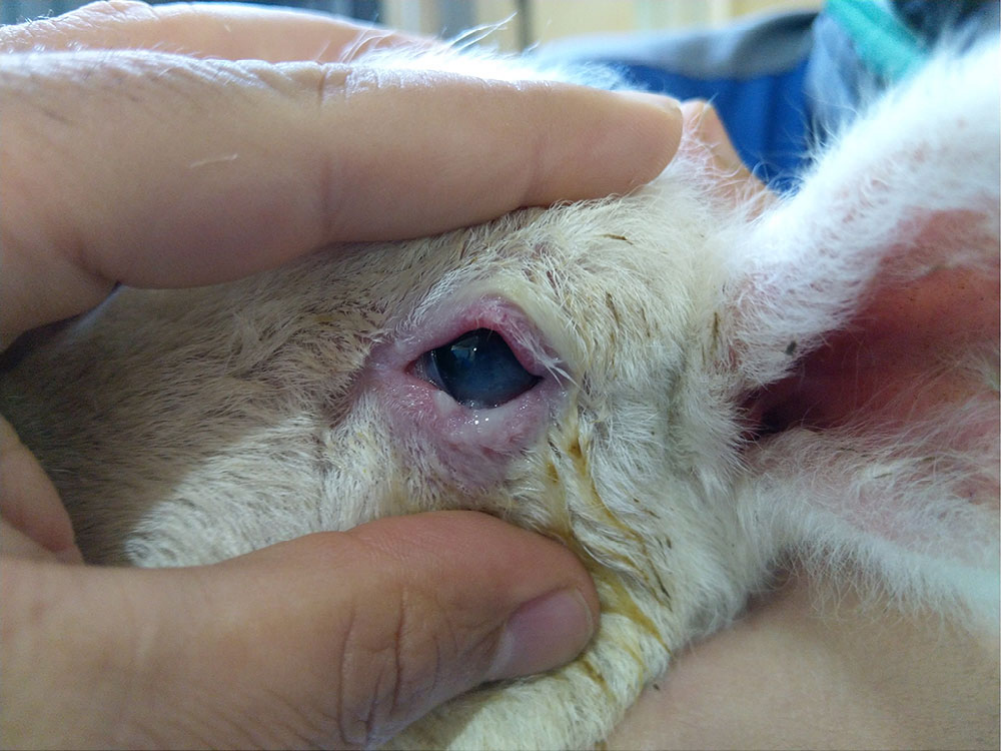
Severe entropion with the presence of ocular redness with conjunctivitis and intense tear discharge and discomfort.

All the studied lambs were examined daily, and the ocular lesion was described as mild or severe based on the following criteria: Mild entropion is characterized by slight inward rotation of the eyelid with occasional contact of the eyelashes with the cornea that did not cause ocular redness or tearing (Figure 1). Severe entropion is characterized by clear inward rotation of the eyelid with severe contact of the skin and eyelashes on the cornea (Figure 2). The final recovery was determined when the lambs were weaned at approximately 90 days of age.

### 2.3 Sampling and bacteriological study

For the microbiological study, samples were taken from 73 affected lambs by sliding a sterile cotton swab over the inner surface of the eyelid and cornea. A sterile medium was used for their conservation, and they were refrigerated and sent as quickly as possible to the laboratory for their microbiological study.

Swabs were inoculated on Blood agar and MacConkey agar (Merck). A pair of plates were incubated aerobically at 37°C, and the other pairs of plates were incubated in anaerobic conditions at 37°C. The plates were observed for bacterial growth after 16–20 h of cultivation, and in cases of no or slow bacterial growth at first inspection, additional observations were performed at 24 h. The selection of colonies for subculturing was based on colony morphology and the number of colony types that were not considered to be post‐mortem contamination. Colonies were subcultured and identified using standard methods for phenotypical characterization (Procop et al., 2020). Mixed cultures containing low numbers of greater than three distinct bacterial colony types with no confirmed pathogenic bacteria were not considered as positive for an aetiologic agent.

### 2.4 Ocular intervention

**FIGURE 3:**
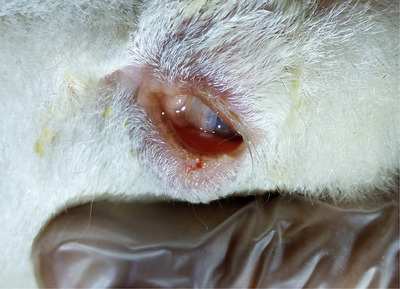
Ocular appearance after antibiotic inoculation.

The ocular intervention was carried out in 123 affected lambs. Each lamb was gently restrained manually for the intervention, whereas no sedatives or general anaesthesia was used. The lambs were prepared for intervention cleaning the eye discharge with a sterile gauze. The ocular intervention consisted of an injection of 0.5–1 cm^3^ of Gentamicin (Razak Company), applied with a 23 gauge 38 mm needle, subcutaneously, in the lower eyelid, approximately 3–5 mm below and along the margin of the entropic eyelid. After injection, tetracycline eye ointment was used daily until entropion was completely resolved (Figure 3).

Lambs were examined daily for entropion recovery status. If it was observed that there had not been a complete recovery, the same intervention was carried out again with the inoculation of an additional 0.5–1 cm^3^ of gentamicin in the lower eyelid. This procedure was performed a maximum of three times. If the entropion was persistent after three interventions, the treatment was suspended. During the study, one lamb died after the first intervention and four after the second.

### 2.5 Statistical analysis

The data were analysed using IBM. SPSS 25 (SPSS Inc.). Categorical data were presented as frequency (percentage). The level of significance was set at *p* < 0.05. Statistical analysis was performed with the Pearson correlation coefficient, Pearson's chi‐square test, Wilcoxon rank‐sum test, Kruskal–Wallis and Fisher's exact test. To report the results in this manuscript, quantitative numbers are shown with the median (IQR).

## 3 RESULTS

Out of the 158 studied lambs with entropion, 35 were from single birth (35/158: 22.1%), 109 were twins (109/158: 68.9%), and 14 were triplets (14/158: 8.9%). In twin lambs, this defect was observed only in one of the twins in 45 cases (45/109: 41.3%), and in 64 cases, both twins were affected (64/109: 58.7%). Although in triplet lambs, a single affected lamb was observed in only one case (1/14: 7.1%), in 11 triple births, two lambs were affected (11/14: 78.6%) and in two cases, the three newborn lambs showed entropion (2/14: 14.3%).

As it was showed in Table 1, the Romane breed developed entropion at an older age (7 days; range 4–12 days) than the Ile de France breed (1 day; range 1–1 day) (*p* < 0.001). In addition, there was a statistically significant difference between bilateral involvement and unilateral (left or right) involvement in all the studied lambs (*p* < 0.001), also when this was analysed in the two different breeds, clear statistical differences were found, bilateral involvement being much more frequent in Ile de France than in Romane breed (71/119: 59.7% vs. 5/39: 12.8%; *p* < 0.001).

**TABLE 1:** Comparison between Ile de France and Romane breed in the age of onset of entropion, eyes affected, development of keratitis and severity of the process.

	Breed			
Variable	Ile de France (*n* = 119)	Romane (*n* = 39)	Total	*p*‐Value
**Age of onset of entropion**				
1–3 Days	110	8	118	
4–14 Days	6	25	31	
> = 15 Days	2	7	9	
Age of affection median (IQR)	1 (range 1–1)	7 (range 4–12)		<0.001
**Eye affected**				<0.001
Both	71	5	76	
Left	23	15	38	
Right	25	19	44	
**Entropion degree**				0.009
Mild	90	37	127	
Severe	29	2	31	
**Keratitis**				0.5
No	78	28	106	
Yes	41	11	52	

Most of the lambs developed mild entropion (127/158: 80.4%), with only 31 (31/158: 19.6%) showing severe lesions. Statistical differences were again found between breeds, showing Ile de France the highest degree of severity (29/119: 24.4% vs. 2/39: 5.1%; *p* = 0.009). However, although keratitis was developed in 52 out of 158 studied lambs (32.9%), no significant differences were found between breeds (Table 1). Nevertheless, the age of onset of entropion was a key factor for the development of keratitis. The older the entropion‐affected lamb was, the less chance it had of developing keratitis, indeed, none of the affected lambs older than 3 months developed keratitis.

No significant differences were found between Ile de France and Romane breeds regarding the final recovery after ocular intervention (103/119: 86.6% vs. 34/39: 87.2%). Spontaneous recovery was observed in 22.1% (35/158) of the affected lambs. However, the Romane breed showed a much greater spontaneous recovery than the Ile de France (23/39: 59.0% vs. 12/119: 10.1%; *p* < 0.001) (Table 2). Of the 35 who recovered spontaneously, 66% (23/35) are of the Romane breed. Likewise, the final recovery day (median; IQR) was 8 (range 5–12 days) for Ile de France and 17 (range 9–25 days) for the Romane breed (*p* < 0.001).

**TABLE 2:** Recovery of entropion in Ile de France and Romane breeds is analysed.

	Ile de France (*n* = 119)	Romane (*n* = 39)	*p*‐Value
**Spontaneous recovery**	12 (10.1%)	23 (59.0%)	<0.001
**After first intervention**	74 (62.2%)	11 (28.2%)	
**After second intervention**	16 (13.4%)	3 (7.7%)	
**After third intervention**	17 (14.3%)	2 (1.7%)	

The average time in which the first ocular intervention was applied in 1–3‐day‐old, 4–14‐day‐old and more than 15‐day‐old lambs was 6 (range 4–9), 10 (range 8–12) and 32 (range 22–42) days, respectively. The final recovery time in these lambs was 8 (range 5–12), 17 (range 10–19) and 28 (range 26–30) days, respectively. The earlier the intervention was performed, the sooner the lambs recovered (*R* = 0.56, *p* < 0.0001). In addition, 63% of the cases with three injections had bilateral entropion and 84% did not recover (*p* = 0.025).

The interval between the onset of entropion and the ocular intervention was significantly related to the development of keratitis (Table 3). In the first intervention, this interval was 4 (range 2–8) days in cases without keratitis and 6 (range 4–10) days in cases with keratitis (*p* = 0.011). The interval of the second and third interventions from the onset of entropion was 11 (range 8–13) and 24 (range 21–30) days, respectively, in cases without keratitis and 32 (range 28–38) and 61 (range 60–62) days, respectively, in cases with keratitis (second injection: *p* < 0.001, third injection: *p* = 0.028).

**TABLE 3:** Development of keratitis related to the day of the ocular intervention.

	Keratitis		
Day of intervention	Yes	No	*p*‐Value
**First injection day** median (IQR)	7 (range 6–12)	6 (range 4–9)	0.029
**Second injection day** median (IQR)	35 (range 29–44)	13 (range 10–20)	<0.001
**Third injection day** median (IQR)	72 (range 71–72)	27 (range 23–31)	0.028

Finally, the bacteria isolated from the ocular swaps taken from 73 cases of entropion included *Staphylococcus* spp. (31/73: 42.5%), *Bacillus* spp. (16/73: 21.9%), *Trueperella pyogenes* (10/73: 13.7%), *Corynebacterium* spp. (9/73: 12.3%) and *Escherichia coli* (7/73: 9.6%).

## 4 DISCUSSION

This is the first study comparing entropion affection between Romane and Ile de France breeds. The median age for the development of entropion in the Ile de France and Romane breeds was 1 and 7 days, respectively, and most of the affected cases above 15 days were Romane (78%). This is the first study to evaluate the onset of entropion, whereas other studies have only argued that entropion is a congenital defect. Most of the complications of entropion happen in young lambs, and usually, keratitis and corneal ulcer are less common in older lambs. Weaned lambs, ewes and rams usually have no keratitis and lacrimation despite entropion.

Congenital entropion in sheep is considered to be a genetic defect, whereas ram selection can successfully decrease its occurrence (Green, 1991; Green et al., 1995; Leipold, 1984). Due to the identification of genes involved in this defect, the transmission of entropion in the flock can be reduced by careful selection when purchasing breeding sheep and by refraining from breeding affected animals.

According to the results of the present study, not all twin and triplet lambs were affected by entropion. Similar to the results of our study, Claine et al. (2013) demonstrated that a number of twins and triplets were not affected by entropion. These reports indicate that although entropion is an inherited defect, it affects the offspring in different degrees. Thus, the severity and extent of its transmission to future generations in different breeds should be further investigated.

Significant differences were found in bilateral or unilateral entropion depending on the breed, being much more common the bilateral disorder in Ile de France lambs. However, there was no significant relationship between left and right eye involvements. Bilateral and unilateral entropion was observed on days 1 and 2, respectively. In the study of Sakul et al. (1996), there was no difference in entropion occurrence between the right and left eyes, and according to the results of the study by Hirter et al. (2020), the incidence of entropion was predominantly observed bilaterally and only some cases had unilateral affection.

Moreover, most severe cases were related to Ile de France breed (5.1% in Romane vs. 24.4% in Ile de France). However, the results showed that the severity of entropion had no significant effect on the final recovery of the animal. Compatible with our results, Sakul et al. (1996) stated that the rate of fully successful treatment was not significantly related to the severity of entropion.

In the present study, 22.1% of cases were recovered spontaneously and the highest rate of spontaneous recovery without injection was observed in Romane breed (66.0%). This may be due to the later onset of entropion in the Romane breed as the median time of entropion onset in the Ile de France and Romane breeds was 1 and 7 days, respectively. Claine et al. (2013) stated that the rate of spontaneous recovery in Texel and Ile de France breeds was 43.7%, and the median time for entropion to recover spontaneously was 7 days (range 2–42 days). Warwick and Berry (1962) reported that most cases of entropion in Rambouillet, Romney Marsh, Cheviots and Silky breeds were recovered spontaneously and only a few cases required surgical correction. Contrary to these results, Wright and Formston (1943) stated in their report that there was no tendency to spontaneous recovery and that corneal thinning and the risk of corneal perforation were common in untreated cases. Spontaneous recovery has been reported in other domestic animals, but most cases require intervention (Holmberg, 1980; Henriksen et al., 2013; Labelle et al., 2011).

In our study, 74% of the cases with an ocular intervention recovered with one injection and 5% did not recover despite three injections. The breed most needing re‐injections were Ile de France. As 63% of cases with three injections have both eyes involved, entropion correction is more important and needs more attention in bilateral cases because it occurs earlier than unilateral cases. Meanwhile, they are more likely to end up in permanent blindness. Wright and Formston (1943) believed that keratocele and progressive corneal necrosis occurred most commonly in entropic lambs if the problem was neglected and not treated. Using a bloodless surgery, Shams‐u‐Din (1992) demonstrated that all entropion cases recovered within 3 days with one intervention. In a study comparing four treatment methods, it was found that excision was the most effective method (92.4%), and subcutaneous injection had 84.3% of efficiency (Sakul et al., 1996). However, in the present study, the efficiency of the subcutaneous injection method was 93.5%. Some other studies also suggest that the injection method is preferred (Green, 1991; Venkataramanan et al., 2009), and undoubtedly, this is the prefer method in farm conditions. The use of a surgical method in the farm has some disadvantages, including the following: It is a time‐consuming operation, sutures often become dirty during suckling and the risk of wound infection increases. Therefore, based on the efficiency of the subcutaneous injection technique, this should be recommended in farm conditions and the recommendation for surgery should be relegated to the few cases in which the subcutaneous injection does not work.

Because of the persistent contact of eyelashes and eyelid skin to the cornea, entropion can lead to keratitis, corneal ulcers and eventually permanent blindness. Our results showed that the lambs that developed keratitis had been intervened on later from the onset of clinical signs than those without keratitis. Therefore, eyelid injections should be given as soon as possible to prevent keratitis. However, based on our experience, we recommend that injections should not be given before 5 days of life because the stress caused may prevent adequate absorption of colostrum immunoglobulins as well as proper bonding between the ewe and the lamb.

The need for re‐injection in Ile de France and Romane breeds was 27.7% and 12.8%, respectively. Although the severity of the entropion was not effective in the final recovery rate after injections, given the rate of spontaneous recovery and the severity of entropion between these two breeds, it is clear that in the absence of treatment, Ile de France is more likely to result in permanent blindness. Various studies have reported the differences in entropion susceptibility among different breeds. According to the results of the study by Claine et al. (2013), Ile de France and crossbred lambs compared to the Texel breed were significantly more affected by entropion. A comparison among the five breeds of Suffolk, Merino, Columbia, Targhee, Rambouillet and Polypay revealed that the Suffolk and Polypay had the highest and lowest incidences of entropion, respectively (Sakul & Kellom, 1997). The association between the entropion and breed was evaluated by Green et al. (1995), who reported that entropion was more prevalent among the Charollais or Texel breeds compared to the Suffolk.

To our knowledge, to date, no study has been conducted on the isolation of bacteria from corneal infections caused by entropion. Similar to our results, *Staphylococcus aureus, Corynebacterium* spp. and *Escherichia coli* were isolated in keratoconjunctivitis cases by Åkerstedt and Hofshagen (2004) in Norwegian sheep. According to the report by Egwu et al. (1989), *Staphylococcus, Bacillus* and *Branhamella* were more frequently isolated than the other genera of bacteria. In the study by Meekins et al. (2017), *Staphylococcus* and *Streptococcus* were the most common bacterial flora found in the conjunctival sac of goats. In regard to the isolation of *Bacillus* and *E. coli*, these two bacteria may have been isolated due to cross‐contamination at the time of sampling. However, their presence in the affected eye can contribute to the deterioration of the eye condition. These florae can be pathogenic in special disorders such as entropion, and they can lead to a worsening of the affected animal. Therefore, the use of antibiotic ointments in entropion cases is required to prevent keratitis and corneal ulcers.

In conclusion, the results of the present study showed that the onset time of entropion, bilateral involvement, the severity of the process and the need for re‐treatment were higher in the Ile de France than in the Romane breed. Likewise, the Romane breed showed a higher degree of spontaneous recovery of entropion. As the Ile de France breed is more susceptible to entropion and entropion treatment is costly and time‐consuming, efforts should be directed to select rams based on their medical history and test results.

## AUTHOR CONTRIBUTIONS

Hossein Esmaeili and Delia Lacasta conceived and designed the experiments; Amir Hossein Alinejad and Mona Hamedi performed the sample collection; and laboratory examination; Hossein Esmaeili analysed the data; Hossein Esmaeili, Sergio Villanueva‐Saz and Delia Lacasta wrote the manuscript. Marta Ruiz de Arcaute, Diana Pérez and Juan Ibañez reviewed the manuscript. Sergio Villanueva‐Saz and Delia Lacasta reviewed the manuscript and corrected the manuscript. All authors read and approved the final manuscript.

## CONFLICT OF INTERESTS STATEMENT

All authors have read and approved the final manuscript. Its contents are solely the responsibility of the authors. All authors declare that they have no conflicts of interest.

## FUNDING INFORMATION

This study was supported by a grant from the University of Tehran (Grant Number:28786).

## ETHICS STATEMENT

Not applicable.

### PEER REVIEW

The peer review history for this article is available at https://publons.com/publon/10.1002/vms3.1317.

## Data Availability

The data supporting this study's findings are available from the corresponding author upon reasonable request.
